# Do Not Skip the Retroflexion: A Case of Disseminated Anorectal Mucosal Melanoma

**DOI:** 10.14309/crj.0000000000000513

**Published:** 2021-02-03

**Authors:** Vibhu Chittajallu, Carlos Roberto Simons-Linares, Olaronke Oshilaja, Prabhleen Chahal

**Affiliations:** 1 Department of Internal Medicine, Cleveland Clinic, Cleveland, OH; 2 Digestive Disease and Surgery Institute, Gastroenterology and Hepatology, Cleveland Clinic, Cleveland, OH; 3 Pathology and Laboratory Medicine Institute, Cleveland Clinic, Cleveland, OH

## Abstract

Less than 4% of melanomas are of mucosal origin, with primary anorectal mucosal melanomas comprising a small subset. Mucosal melanomas are often diagnosed at an advanced stage due to delay in patient presentation and obscured tumor origins leading to a more aggressive behavior and less favorable prognosis when compared with cutaneous melanomas. We present a case of metastatic anorectal mucosal melanoma with a negative colonoscopy 1 year earlier.

## INTRODUCTION

Colonoscopy is a highly sensitive and specific screening modality for colorectal cancer detection.^1^ In addition, this procedure allows not only for detection but also resection of most precancerous polyps and neoplastic lesions.^1^ Unlike colon cancer, which is the third most common malignancy globally, melanoma accounts for only 4%-7% of total malignancies and is the fifth most frequent malignancy in the world.^2,3^ Melanoma is further subdivided into cutaneous and mucosal type. Mucosal melanomas are rare and arise primarily in the head and neck, anorectal, and vulvovaginal regions. Anorectal mucosal melanoma (AMM) is an extremely rare entity, and there is a significant paucity of literature on this malignancy.^4^ AMM most often affects females in the fifth to seventh decade of life, and rectal bleeding is the most common chief complaint.^5^ AMM more often affects the rectum than the anal canal; however, the 2 entities usually are studied together.^6^ The significance of mucosal melanomas when compared with cutaneous melanomas lies in their propensity to be more aggressive with less favorable outcomes.^4^ Approximately 20%-70% of patients with AMM present with metastases at the time of diagnosis, with a dismal 5-year survival rate of 10%-20%.^7^ We present a case of AMM diagnosed by retroflexion maneuver performed during repeat colonoscopy in a female patient presenting with vague gastrointestinal symptoms with a normal digital rectal examination (DRE) and a normal colonoscopy within the past year.

## Case report

A 68-year-old woman presented to the hospital with a history of several months of intermittent fecal impaction complicated by sporadic outlet type rectal bleeding, a negative screening colonoscopy 1 year earlier, and a normal DRE 2 months earlier. She endorsed progressive anorexia and a 20lb weight loss over the past few months. On presentation, she was hemodynamically stable with a normal hemoglobin level. Computed tomography imaging depicted multiple lesions in the liver and lungs concerning for metastatic disease.

**Figure 1.**
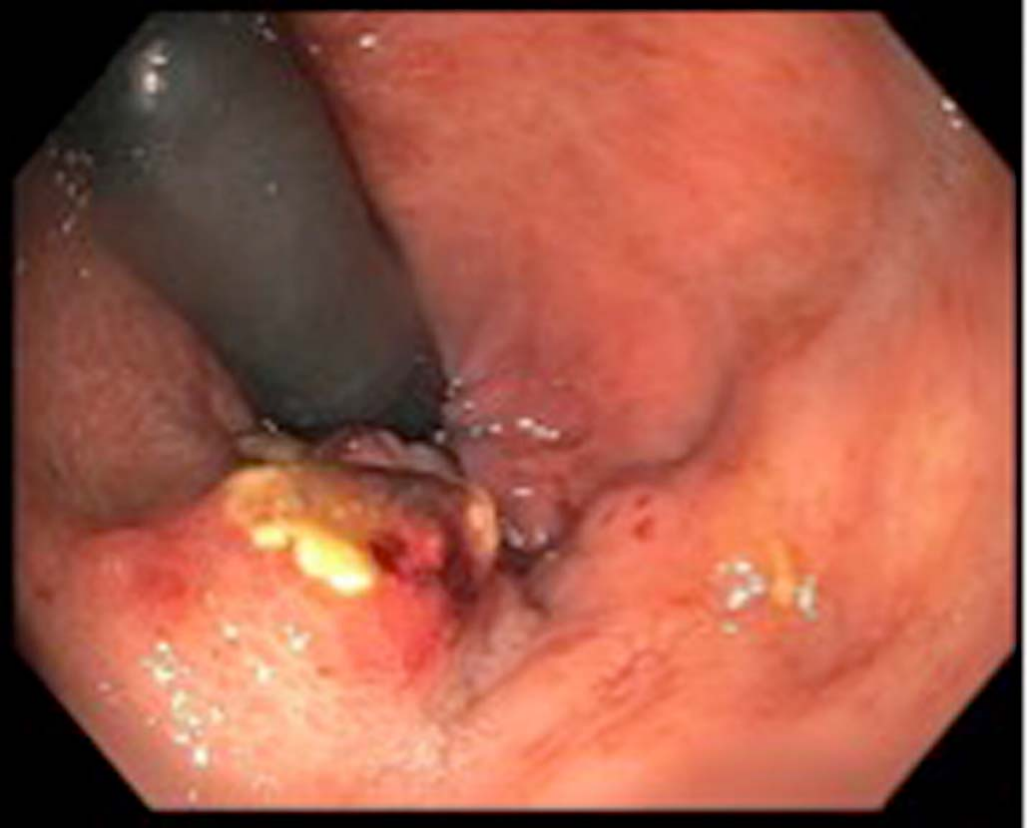
Colonoscopy demonstrating rectal mass on retroflexion.

**Figure 2.**
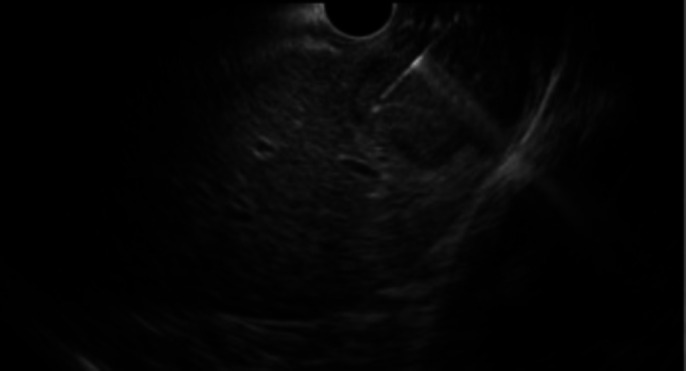
Endoscopic ultrasound showing metastatic liver lesions.

**Figure 3.**
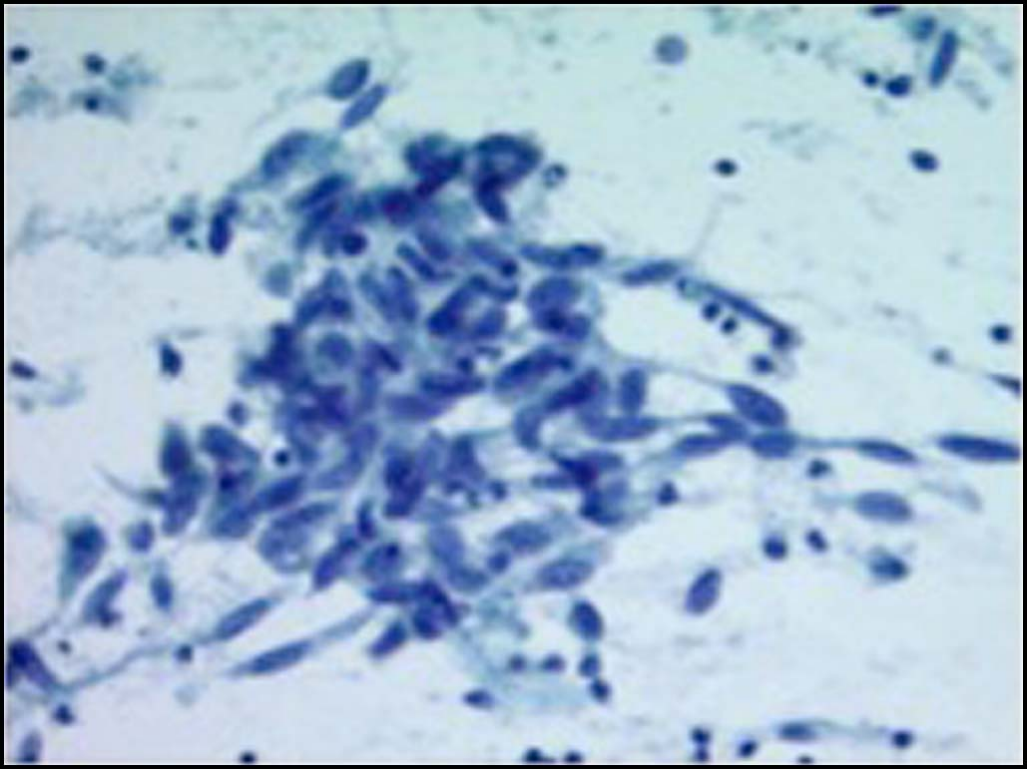
Liver lesion fine need aspiration smear demonstrates a loosely cohesive group of hyperchromatic spindle malignant cells (Papanicolaou stain, 20×).

During repeat colonoscopy, a rigid mass was felt on rectal examination and granular nodularity with ulceration in the rectum extending up to the dentate line was visible on retroflexion (Figure 1). A biopsy confirmed mucosal melanoma. Same-session endoscopic ultrasound-guided fine-needle aspiration of left hepatic lobe lesions confirmed malignant melanoma (Figures 2 and Figure 3). Staging magnetic resonance imaging revealed primary tumor involvement of the anal canal and rectum in addition to metastases to the liver, lungs, and abdominal lymph nodes.

The patient was diagnosed with T4N1M1, stage IV AMM (BRAF/C-KIT negative), and was recommended to undergo ipilimumab and nivolumab (I3/N1) immunotherapy with palliative radiation. The patient completed 4 cycles of ipilimumab/nivolumab and a total of 2000 Gy in 5 fractions of radiation therapy without complication. Repeat imaging demonstrated a reduction in primary tumor and metastatic disease. She was continued on monthly maintenance nivolumab therapy. Patient still endorses symptoms of constipation with resolution of rectal bleeding.

## DISCUSSION

Anorectal mucosal melanoma is a rare malignancy with a known poor prognosis. AMM's less favorable prognosis is believed to be related to delay in diagnosis because of late symptom presentation and from obscured tumor origins.^8^ Despite these setbacks, a good-quality colonoscopic examination is imperative in diagnostic evaluation of this entity.

A standardized, high-quality colonoscopic evaluation includes a DRE and retroflexion before endoscope withdrawal. DRE is an underused physical examination maneuver both inside of and outside of the endoscopy suite that can be used to assess not only for anorectal masses but also perineal sensation, anorectal function, and rectoceles.^8^ In addition, retroflexion in the rectum serves a fundamental purpose in adenoma detection with more than 50% of all lesions in the distal rectum only being identified on retroflexion.^9^ The importance of these key steps in clinical evaluation of a patient presenting with unknown rectal bleeding is highlighted in this case report.

Diagnosis of AMM is difficult with endoscopic appearance demonstrating ulcerated polypoid lesions with irregular surfaces and generally amelanotic but sometimes with brown/black pigmentation.^6^ In addition, on immunohistochemistry, AMM normally shows a strong positivity to S100, Melan-A, tyrosinase, and HMB-45, while usually negative for pan-cytokeratin.^6^ Once diagnosed, treatment options for AMM are only moderately successful with surgery being the cornerstone of treatment. Surgical options include wide local excision (WLE) or radical excision (abdominal perineal resection [APR]); however, there are no significant differences in survival between WLE and APR.^11^ WLE offers a much less morbid operation, while APR may offer a higher rate of local control.^10^ Finally, rate of locoregional recurrence is improved in APR (42%) compared with WLE (71%).^10^ Survival after surgical intervention significantly decreases with advanced staging. In addition to surgical intervention, treatment for mucosal melanoma includes c-KIT(imatinib), CTLA-4 (ipilimumab), and PD-1 inhibition (nivolumab) therapies.^11^ The objective response rate (ORR) is superior for PD-1 inhibition (ORR 35%) over CTLA-4 inhibition (ORR 10%) as single agents for mucosal melanoma; moreover, combination therapy has had promising response rates for cutaneous melanoma to be considered for mucosal melanoma.^12^

AMM is a deadly diagnosis that portends a poor prognosis because of it frequently being diagnosed late as advanced metastatic disease.^10^ High-quality colonoscopy is imperative in diagnosing this malignancy with strict adherence to standardized endoscopic evaluation protocols. This case is a prime example of not only the stealth and aggressive nature of anorectal mucosal melanoma presenting with disseminated disease despite normal colonoscopy 1 year earlier but also of the importance of the rectal examination and careful endoscopic retroflexion maneuver during colonoscopy.

## DISCLOSURES

Author contributions: V. Chittajallu wrote the manuscript and reviewed the literature. CR Simons revised the manuscript for intellectual content and provided the figures. O. Oshilaja provided the figures. P. Chahal revised the manuscript for intellectual content and is the article guarantor.

Financial disclosure: None to report.

Previous presentation: This case was presented at the Ohio Gastroenterology Society Annual Meeting, September 14, 2019; and the American College of Gastroenterology Annual Scientific Meeting, October 25-30, 2019; San Antonio, Texas.

Informed consent was obtained for this case report.
